# Low-Cost Self-Driven Liquid Biosensor Based on Metamaterials for Glioblastoma-Related Sample Detection

**DOI:** 10.3390/bios16090476

**Published:** 2026-08-30

**Authors:** Kanglong Chen, Minghui Du, Peiyuan Sun, Pei Yang, Xiaojun Wu

**Affiliations:** 1 Hangzhou International Innovation Institute, Beihang University, Hangzhou 311115, China; klchen@buaa.edu.cn; 2 Department of Neurosurgery, Beijing Tiantan Hospital, Capital Medical University, Beijing 100070, China; minghuidu@mail.ccmu.edu.cn (M.D.); spy_ttyy@mail.ccmu.edu.cn (P.S.); 3 Beijing Neurosurgical Institute, Capital Medical University, Beijing 100070, China; 4 School of Electronic and Information Engineering, Beihang University, Beijing 100191, China; 5 Zhangjiang Laboratory, 100 Haike Road, Shanghai 201210, China

**Keywords:** terahertz metamaterials, liquid biosensor, suspension, serum, glioblastoma detection

## Abstract

A self-driven terahertz metamaterial liquid biosensor composed of channel-structured metamaterials and a quartz microcavity is proposed for glioblastoma sample detection. The device transports liquid samples via capillary force. The permittivity (*ε_eff_*) of liquid suspensions is comprehensively analyzed to provide theoretical support for the detection. For suspensions with the same contents, *ε_eff_* decreases with increasing concentration, while under the same concentration condition, *ε_eff_* decreases as particle size grows. An electric dipole resonance is excited at ~1.54 THz with theoretical sensitivity ≥ 242 GHz/RIU (where RIU denotes refractive index unit). For the same type of cell discrimination, the frequencies of the biosensor’s feature peaks shift from ~1.14, ~1.18, and ~1.20 THz with the rise in cell concentration of glioblastoma stem cell (GSC) suspension from 4 × 10^5^, 6 × 10^5^ to 8 × 10^5^ cells/mL, respectively. In addition, the GSC, U87 and U251—whose average diameters increase in that order—tested at the same concentration of 4 × 10^5^ cells/mL lead to feature peak shifts from ~1.14, ~1.17, and ~1.20 THz. Clear THz differences exist between healthy and patient serum, with peaks at 1.18 and 1.19 THz. The sensor effectively distinguishes cell suspensions but has limited serum discrimination capacity. The sensor retains cell morphology, requires little pretreatment, and is low-cost and fast for rapid glioblastoma clinical screening.

## 1. Introduction

Gliomas are primary intracranial tumors originating from glial cells of the central nervous system, and are the most common intracranial tumors in adults [1,2]. Glioblastoma, IDH-wildtype, is classified as CNS WHO grade 4 and represents the most aggressive diffuse glioma in adults. Despite advances in multimodal treatment, including maximal safe surgical resection followed by radiotherapy and temozolomide chemotherapy, the prognosis remains poor, with a median overall survival of approximately 14.6 months [3]. Grade IV glioma, also called glioblastoma (GBM), is the deadliest subtype. Approximately 95% of GBM patients have a survival time of no more than five years. Among GBM cell lines, U87 and U251 are long-term-passaged, differentiated, adherent tumor cells with stable genetic backgrounds. They are commercially available. GSCs possess strong tumorigenicity and drug resistance, which reflect the clinical pathological features of tumor recurrence, invasion and treatment resistance. GBM continually generates tumor secretions, leading to significant differences between patient serum and healthy serum. The traditional methods used to distinguish these three types of cells—including flow cytometry, Western blotting, and qRT-PCR—typically require 4–24 h of sample processing, entailing lysis, labeling, or target amplification steps [4,5,6]. Such methodologies necessitate costly antibodies or fluorescent reagents, dedicated analytical instrumentation, and trained technical personnel, and native cellular morphology is irreversibly disrupted throughout sample preparation. For serum-based glioma detection, analytical platforms targeting soluble proteins, exosomes, and circulating nucleic acids are each associated with well-documented limitations: protein biomarkers such as GFAP exhibit restricted diagnostic specificity; exosome isolation procedures are labor-intensive and cost-intensive; and nucleic acid-based assays demonstrate limited sensitivity for early-stage tumor identification [1,7,8,9]. Collectively, existing detection modalities remain constrained by inherent trade-offs among diagnostic specificity, operational cost, procedural complexity, and analytical sensitivity. Hence, it is imperative to develop a fast, low-cost, straightforward, and sensitive biosensor for the identification of these cells and serum samples.

Fortunately, biosensors based on THz metamaterials (MMs) can satisfy these detection requirements. MMs are artificial periodic structures composed of repeating unit cells. Each unit cell consists of a substrate and parts with metal shapes. MMs-based terahertz (THz) biosensors have attracted widespread attention owing to their promising applications in biological detection [10,11,12,13]. When excited by incident terahertz radiation, electromagnetic energy is tightly confined within deep-subwavelength near-field volumes by the unit cell, giving rise to pronounced localized electric field enhancement. When an analyte is introduced into the near-surface sensing region of the MMs, the effective permittivity of the local dielectric environment is changed, which in turn induces measurable modulations in the transmission spectra of MMs. Through quantitative monitoring of these spectral responses, label-free discrimination of analytes with distinct biochemical compositions or varying concentration levels is enabled by the metamaterial sensing platform.

In recent years, numerous THz biosensors based on MMs have been reported. A triple-layer metamaterial biosensor has been deployed for exosome-based early colorectal cancer diagnosis, with analytes dried onto the chip surface prior to spectral characterization [14]. Likewise, biosensors based on double split-ring resonators and quasi-bound states in the continuum (Q-BIC) have been proposed for tumor antigen detection and low-concentration protein solution analysis, the majority of which likewise rely on dried analyte films deposited on the metamaterial surface [15]. While these configurations deliver favorable sensitivity performance, their multi-layer architectures or high-resolution resonant patterns incur elevated fabrication complexity and cost. More critically, the requisite drying procedure increases operational overhead and assay turnaround time, and removes analytes from their native aqueous environment, such that intact cellular morphology and physiological state cannot be preserved throughout measurement. For liquid-phase detection, flexible polyimide (PI)-capillary-integrated Q-BIC sensors have been experimentally demonstrated [16]. Nevertheless, the compliant polymer substrate is susceptible to mechanical deformation under repeated manipulation, which introduces uncontrolled variations in confined liquid volume and degrades measurement reproducibility. An alternative microcavity-integrated metamaterial sensor has enabled label-free discrimination of multiple suspension cell lines, yet performance validation has been restricted to commercially available cell lines, with no extension to clinically relevant specimens such as patient-derived serum [17]. Collectively, pronounced limitations remain inherent to existing THz metamaterial biosensors when translated toward practical clinical glioblastoma screening workflows. Dry-state sensing modalities cannot preserve native biological characteristics, and necessitate cumbersome sample preparation protocols. Existing liquid-phase designs, by contrast, are constrained by either insufficient structural robustness or limited analyte compatibility. Few sensing platforms have been concurrently validated against both primary tumor cells and clinical serum specimens. Accordingly, a clear unmet need persists for a low-cost, robust liquid-phase THz biosensor with facile fabrication, capable of assaying both cellular and serum specimens for rapid glioblastoma detection.

Here, a self-driven microfluidic terahertz biosensor is proposed. The device consists of channel-structured metamaterials and a quartz microcavity, and enables parallel detection of cellular suspensions and serum specimens. The microcavity consists of two quartz layers: the upper layer is patterned with microfluidic channels, and the lower layer serves as the supporting substrate. Channel-shaped apertures are likewise engineered into the metamaterial unit cells. Upon assembly, a closed microfluidic network is formed between the microcavity and the metamaterial layer, where capillary forces drive spontaneous, uniform wetting of the sensing region without external pumping actuation. The metamaterial architecture supports a fundamental electric dipole bright mode at ~1.54 THz, with a theoretically determined minimum refractive index sensitivity of no less than 242 GHz/RIU. Based on an effective permittivity model for ideal suspensions, the combined effects of particle concentration and size on bulk suspension permittivity are analyzed, establishing a theoretical framework for interpreting experimental spectral trends. Experimental validation is conducted across multiple sample panels. Concentration-dependent responses are verified for glioblastoma stem cell (GSC) suspensions across three concentration levels. Differentiation among GSC, U87, and U251 cell lines is further confirmed at a matched number concentration. For clinical serum specimens, measurable spectral discrepancies are observed between healthy donors and patients with grade IV glioblastoma. Limitations of the biosensor—including limited resolution between serum samples from individual patients of identical pathological grade and the absence of full statistical calibration—are discussed alongside targeted optimization strategies. This work delivers a low-cost, label-free, and rapid liquid-phase sensing architecture with potential utility as an adjunctive modality for glioblastoma screening.

## 2. Design and Theoretical Foundation

### 2.1. Biosensor Design

**Figure 1:**
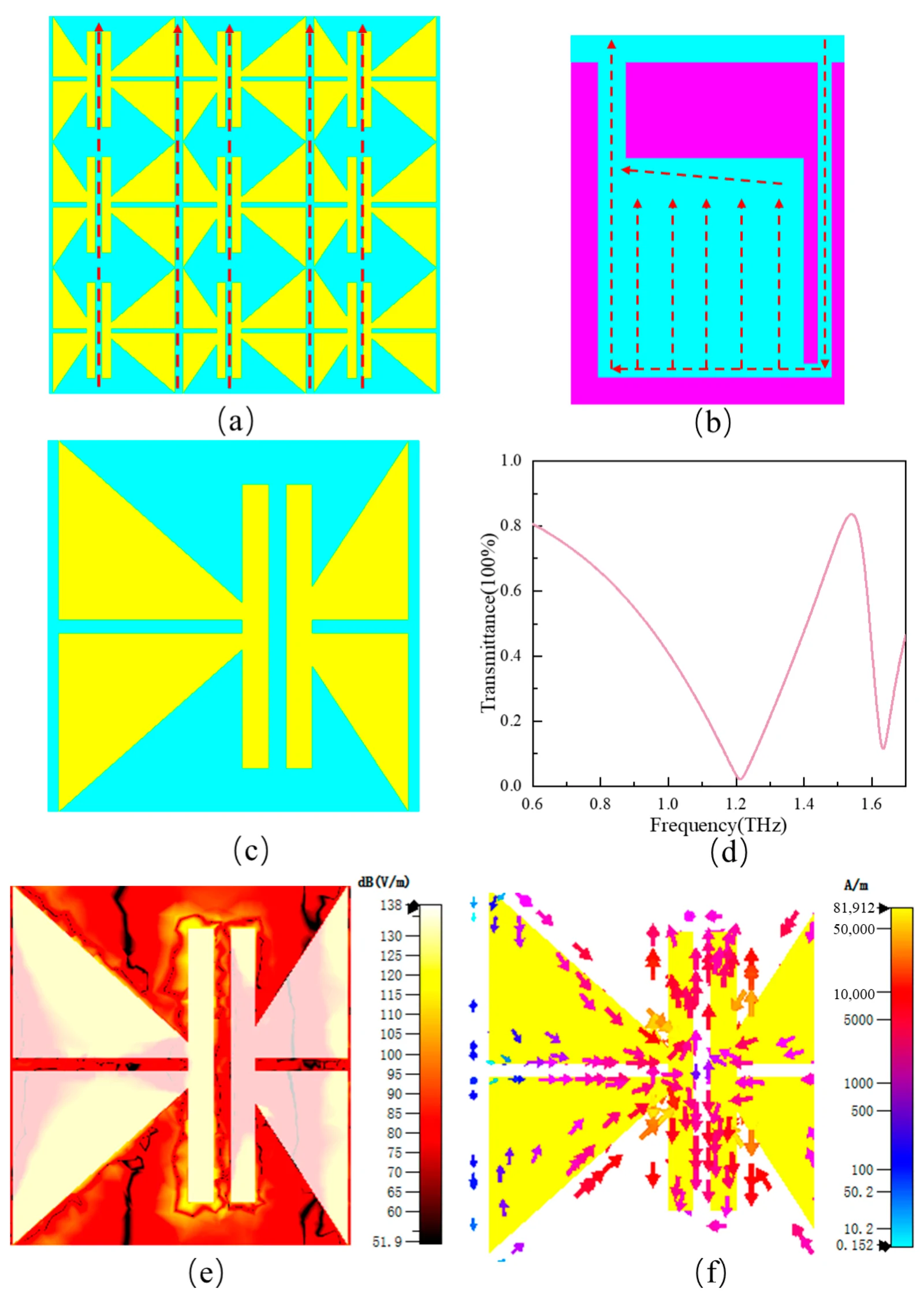
The proposed biosensor. (**a**) The array and the direction of liquid flow. (**b**) The microcavity and direction of liquid flow. (**c**) The unit cells. (**d**) The simulation transmittance curve. (**e**) Electric field and (**f**) surface current distributions at ~1.52 THz.

The liquid biosensor consists of metamaterials (MMs) and a microcavity. Figure 1a displays the typical unit array of metamaterials. The yellow metallic patterns represent microchannels used to guide liquid along the marked direction indicated by red arrows. As presented in Figure 1b, the microcavity is made up of two quartz (JGS2, China) components, including a blue substrate and a magenta structural layer. The magenta section forms microchannels which drive liquid along the path of the red arrows [18]. Detailed geometric parameters of the unit cell and the microcavity are provided in the Supplementary Materials, as shown in Figure S1.

When the electric field component of the incident terahertz radiation is polarized parallel to the channel orientation of the unit cell, the numerically calculated transmittance curve ranges from 0.6 to 1.7 THz, as shown in Figure 1d. A resonance window appears from ~1.22 to 1.62 THz, with its peak at ~1.54 THz. This resonance is selected as the feature peak of the biosensor. A normalized electric field distribution is given in Figure 1e to explain the underlying physical mechanism at 1.54 THz. The electric field is strongly enhanced and confined at the terminals of the vertical metal strips. Such local field enhancement originates from charge accumulation at the strip ends, which is a typical feature of electric dipole resonance [19]. The surface current distributions shown in Figure 1f can further reveal the underlying physical mechanism. The surface current distribution shows unidirectional current flow along the length of the metallic bars. The current reaches its maximum magnitude at the center of each bar and decreases toward the ends. This current pattern corresponds to the collective oscillation of free electrons along the bars which drives the electric dipole resonance [20]. No closed-loop currents or quadrupole-like field patterns are observed. Thus, the dominant resonant mode at this frequency is identified as the fundamental electric dipole mode. The mode is directly coupled to the incident electromagnetic wave and classified as a bright mode [21,22].

### 2.2. The Permittivity of Suspension

In prior work, the effective permittivity (*ε_eff_*) of suspensions has been thoroughly analyzed [17]. In the terahertz regime, the liquid suspension analyte is treated as an ideal suspension model. Cells and proteins do not exchange substances with the solvent. In other words, the shape and diameter remain unchanged during the measurement period, which means the cells and protein in the suspension are regarded as rigid particles. The *ε_eff_* can be described as follows [23]:

(1) $$\varepsilon_{eff}=\varepsilon_{w}+(\varepsilon_{p}-\varepsilon_{W})V$$

Here, *ε_w_* and *ε_p_* denote the dielectric constants of the water and the suspended particles, respectively. V is the dimensionless volume fraction of particles in the suspension, defined as *V* = *N* · (4π/3)*d*^3^, where *N* represents the particle number concentration (number of particles per unit volume of suspension), and d is the radius of an individual spherical particle. The term (4π/3)*d*^3^ corresponds to the volume of a single particle; multiplying this value by the number concentration *N* yields the total volume occupied by particles per unit volume of suspension. In suspensions, the *d* of cells are on the micrometer scale; the *d* of protein ranges from 3 to 6 nm, while the *d* of the ion (the Na^+^ (~0.37 nm), K^+^ (~0.32 nm), Cl^−^ (~0.34 nm), HPO_4_^2−^ (~0.6 nm), and H_2_PO_4_^−^ (~0.6 nm) in PBS solution) (detailed information regarding the PBS solution is presented in the Supplementary Materials) is below 1.5 nm. The *d*_ion_ is far smaller than the *d_cell_* and *d_protein_*. Hence, the *ε_eff_* of the suspension mainly depends on the d and *N* of the cells and proteins.

The working frequency of the liquid biosensor ranges from 0.6 to 1.7 THz. In this frequency range, the permittivity of pure water is 5.7 < *ε*_w_ < 6.2, the permittivity of the cell is *ε_cell_* < 4.5, and the permittivity of the protein is *ε_protein_* < 4.3 [23,24,25,26,27,28,29]. Therefore, *ε_p_* − *ε_w_* < 0 holds for all biological particles considered in this work. From Equation (1), a larger *V* reduces the overall effective permittivity *ε_eff_*. Two trends follow directly: (a) For suspensions of the same particle type (fixed *d*), a rising concentration based on *N* increases *V* and therefore decreases *ε_eff_*. (b) For suspensions under the same concentration *N* condition, a larger particle radius d increases individual particle volume, which also raises *V* and accordingly reduces *ε_eff_* [17]. The dependence of εeff on cell/protein diameter and number-based concentration induces shifts in the metamaterial transmission spectrum.

### 2.3. Theoretical Sensitivity Calculation

At ~1.54 THz, when the polarized electric field of the incident THz wave is parallel to the channel, a bright mode can be excited. The bright resonant mode offers metamaterial sensors strong, easily detectable resonant signals. The sensitivity of the biosensor based on this structure has been numerically calculated. In this work, Δ*f* denotes the absolute resonance frequency shift relative to the bare MMs. The theoretical sensitivity is defined as *S* = Δ*f*/Δ*n*, the frequency shift per unit change in refractive index, so the unit of *S* is GHz/RIU (RIU, Refractive Index Unit) [30,31,32].

**Figure 2:**
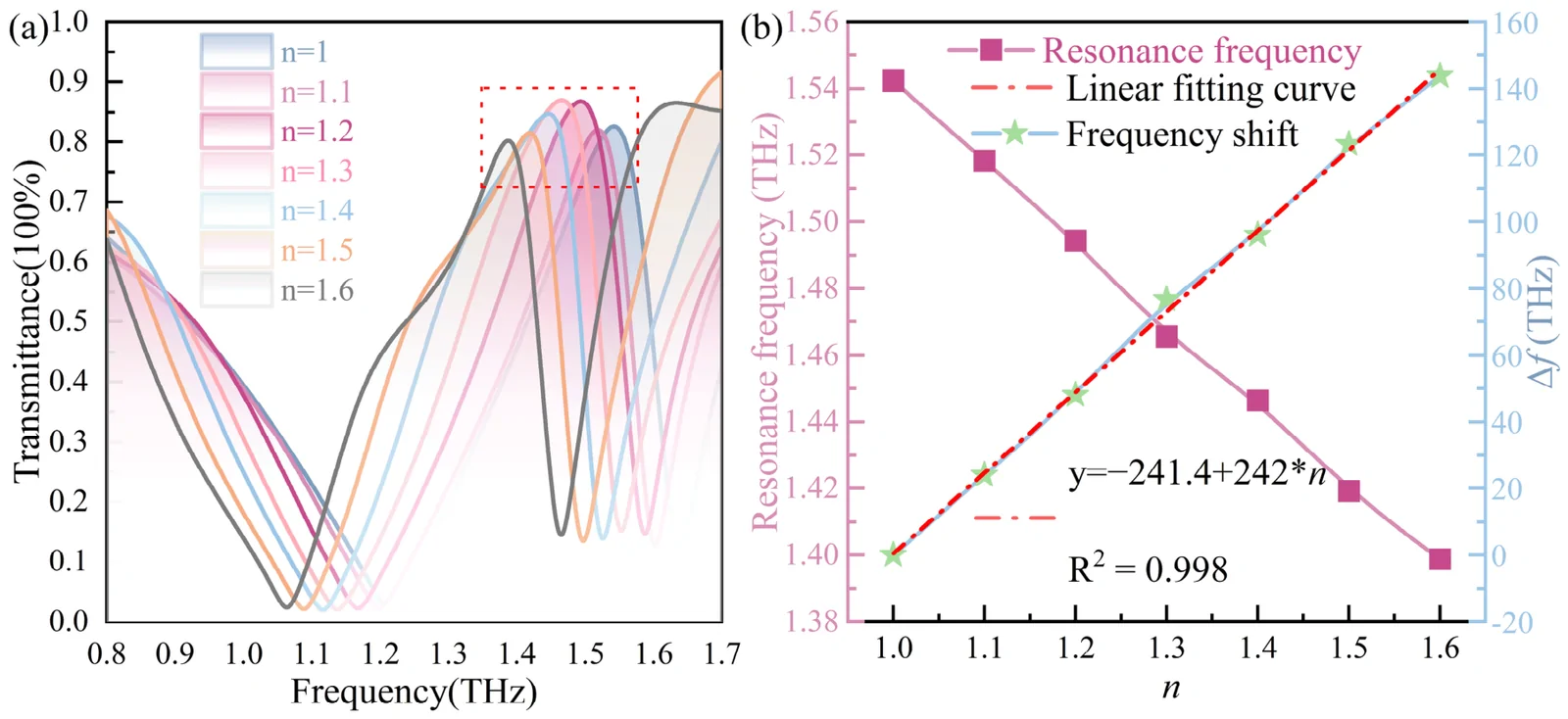
The sensitivity calculation results. (**a**) Transmittance curves based on various *n* of the self-defined analyte. (**b**) Resonance frequency shifts Δ*f* extracted from (**a**) and the corresponding linear fitting curve.

A kind of self-defined analyte was deposited onto the MMs; also, the gap in the metal shape is fully filled in by the analyte. The analyte’s permeability *μ* = 1, so the permittivity ε = *n*^2^; *n* is the refractive index of analyte, and *n* ranges from 1 to 1.6. The analyte thickness is identical to that of the liquid layer in the microcavity. Figure 2a presents transmittance curves depending on the various n values. The feature peak (enclosed within the dashed box) redshifts from ~1.54 THz to ~1.40 THz as the refractive index increases from 1 to 1.6. Figure 2b shows the linear relationships of feature peak resonance frequency and Δ*f* with the refractive index. Both the resonance frequency curves and frequency shift curves are almost linear. The absolute value between each feature peak is not less than 24 GHz. The linear fitting curve of the frequency shift is presented, with a slope of ~242, and linear regression of the frequency-shift data yields a coefficient of determination R^2^ > 0.998, confirming a highly linear response across the tested refractive-index range. This indicates that the theoretical refractive index sensitivity of the designed MMs is not less than 242 GHz/RIU. According to the theoretical sensitivity, the MMs are suitable for biosensing applications. 

## 3. Method

### 3.1. The Suspension Preparation

In this work, six types of suspension were measured using the proposed liquid biosensor, as listed in Table 1.

Samples from S1 to S6 were prepared with different suspensions. Sample S1 was a glioblastoma stem cell (GSC) suspension. S2 and S3 were GBM cell suspensions. S4, S5, and S6 corresponded to Serum1, Serum2, and Serum3, respectively.

### 3.2. Cell Suspension Preparation

The GSCs (BNI-2-4) were obtained from glioma patients (Beijing Tiantan Hospital, Capital Medical University (BJTTH), Beijing, China). The GBM cells were commercial GBM cells (National Infrastructure of Cell Line Resource, Beijing, China), and the contents in S2 and S3 are U87 and U251, respectively. Both GSC and GBM cells were cultured using a standard protocol [33]. Once these cells were cultured to adhere to the surface of culture dishes, cells were rinsed with PBS; this was followed by digestion with trypsin–EDTA solution. After digestion, the cells were centrifuged, collected, and subsequently re-rinsed with PBS [17]. Finally, the cells were prepared in suspensions at specific concentrations. The cell number concentration *N* in each suspension was quantified using a cell counter (NucleoCounter^®^ NC-200™, ChemoMetec, Allerød, Denmark; details are provided in the Supplementary Materials). Each suspension was measured immediately after preparation. At least three independent replicate measurements were carried out.

### 3.3. Serum Suspension Preparation

Serum sample S4 was obtained from a healthy volunteer (Minghui Du, the second author), and serum samples S5 and S6 were acquired from two patients with stage-IV glioma (BJTTH, Beijing, China). Once the blood was obtained, specimens were transferred into anticoagulant-free containers and maintained in an upright orientation at 22 ± 2 °C for a fixed 45 min interval to allow complete clotting. The 30~60 min timeframe cited in the literature constitutes a widely accepted clinical window for this procedural step. A uniform 45 min clotting protocol was implemented for all samples in this study to ensure experimental consistency. After that, centrifugation was carried out at 3000 r/min for 10 min. The pale yellow supernatant was gently aspirated to obtain serum. Each serum sample was divided into no fewer than three aliquots as required. Similarly, measurements were performed immediately after serum preparation. 

### 3.4. Liquid Biosensor Preparation

The liquid biosensor consists of a microcavity, MMs, and a holder. The complete microcavity structure is fabricated by bonding the structural layer onto the quartz substrate, and the final biosensing chip is assembled by integrating the metamaterial sheet with the prefabricated microcavity. Given that the metallic resonant pattern on the metamaterial has a thickness of only 200 nm, the cavity height of the biosensing chip is directly dictated by the thickness of the structural layer. Accordingly, the liquid analyte layer confined within the microcavity exhibits a nominal thickness of approximately 200 μm. This thickness is rationally chosen to ensure that incident terahertz radiation penetrates the liquid layer with acceptable attenuation, while enabling efficient excitation of metamaterial resonance for analyte detection [34,35]. These accessories were cleaned before the sensor was used for measurement. Firstly, these accessories were washed in ultrasonic cleaners (UC5431, Stanford Advanced Materials, Lake Forest, CA, USA) with deionized water. Subsequently, these accessories were rinsed with 75% ethanol. After that, the components were dried under a nitrogen environment at 37 °C. The procedures for biosensor assembly and liquid sample transfer are shown in Video S1 (Supplementary Video). In the video, the pipette (Eppendorf, Hamburg, Germany) is used to transfer liquid. The atmospheric pressure can make two polished quartz plates bind with each other when liquid remains on the surface [36,37,38,39]. This effect prevents liquid leakage.

After the suspensions and serum were prepared, the liquid biosensor was assembled. All samples were measured by terahertz time-domain spectroscopy (THz-TDS). The frequency resolution of the THz-TDS system is 5 GHz [40,41,42,43]. Detailed parameters of the testing system are given in the Supplementary Materials, as shown in Figure S2. The fast delay line technology was adopted in the measurement, and the delay line was fixed at 30 mm [44,45]. As a result, reflection interference was generated in the measured data.

## 4. Measurement and Analysis

### 4.1. The MMs and Liquid Biosensor Verification

**Figure 3:**
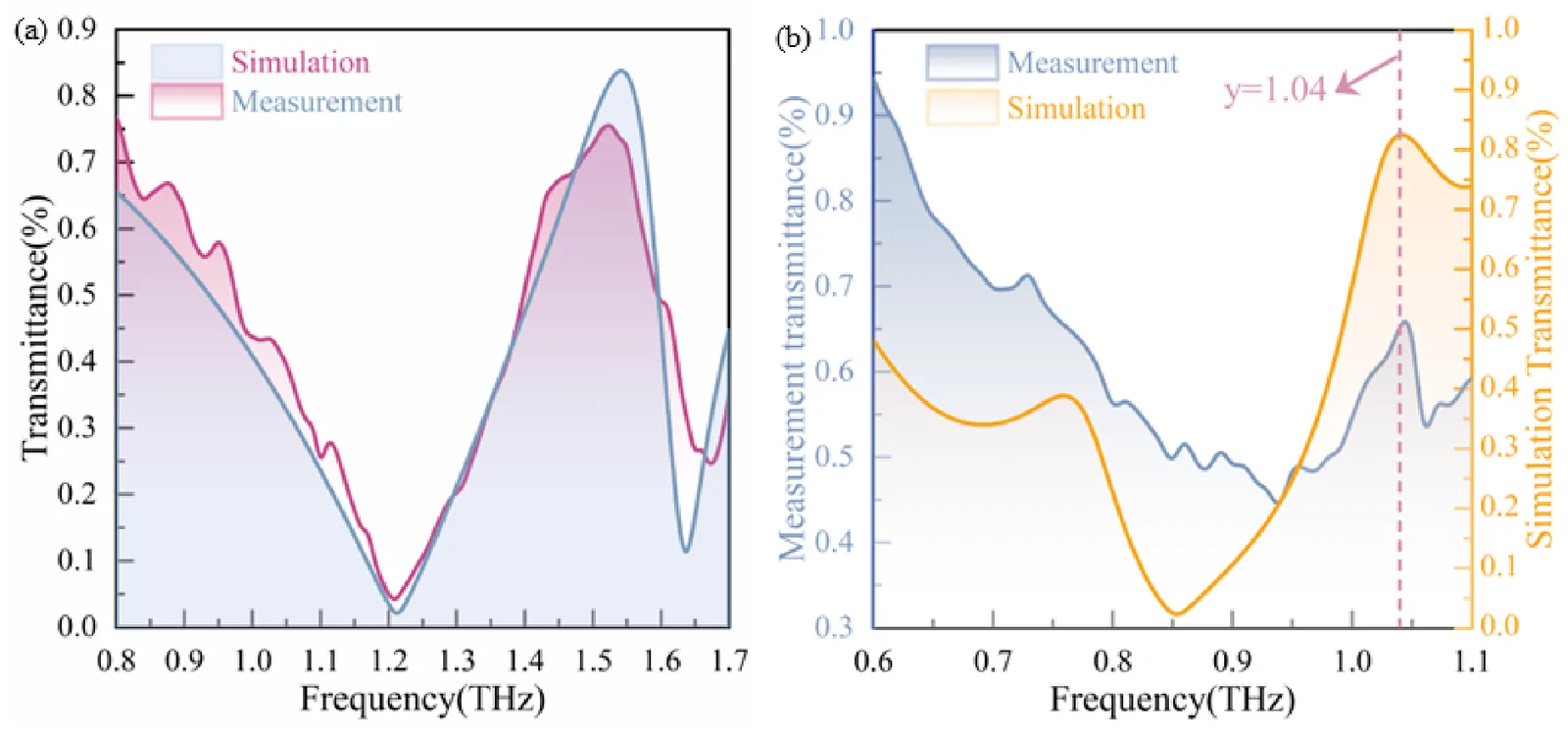
Comparison of results. (**a**) Simulated and measured transmittance curves of metamaterials. (**b**) Simulated and experimental results of water samples.

To verify the sensor performance, the MMs and assembled liquid biosensor were measured using THz-TDS. In this work, MMs were custom fabricated via conventional photolithography by Henan Weina Semiconductor Technology Co., Ltd. (Luoyang, Henan Province, China). A standard fabrication workflow was adopted, consisting of photoresist spin-coating, photomask-based UV exposure, development, gold thermal evaporation, and lift-off. The final gold pattern was fabricated with a thickness of 200 nm on a 500 μm thick JGS2 quartz substrate. Figure 3a presents the measured and simulated results of the MMs. The envelope of the measured curve agrees well with the simulated curve, given the presence of ripple oscillations. In the measurement, the scan time was ~200 ps. Both the MMs and the microcavity substrates have a thickness of 500 μm. Interfaces between the MMs/microcavity structures and the surrounding air/liquid media give rise to interfacial reflections. In addition, for fused silica, which has a refractive index of ~1.95 in the terahertz band, a thickness of 500 μm yields a Fabry–Pérot (FP) round-trip time delay of ~6.5 ps. The 200 ps time-domain scanning window captures dozens of such successive reflection echoes. Collectively, the interfacial reflections and the FP effect produce the ripple oscillations observed on the transmission curves (details regarding the impact of ripple waves on sensor sensitivity are provided in the Supplementary Materials) [46,47]. In addition, machining errors lead to slight deviations in transmittance amplitude and resonant frequency between experiment and simulation. Such minor differences are acceptable for this work.

In the range of 0.6~1.3 THz, the permittivity of pure water is 5.7 < *ε_w_* < 6.2. Hence, a water layer with a thickness of ~200 μm and *ε_w_* = 6 was selected as the analyte, and covered the biosensor surface [17,48,49]. In the measurement, the reference signal was obtained from a water-filled microcavity assembled with a bare metamaterial substrate. The numerically calculated transmittance curve demonstrates that the feature peak shifts to ~1.04 THz. It is worth noting that the imaginary part of *ε_w_* was not considered, so the dielectric loss of water was ignored. (The effect of the imaginary part of *ε_w_* on sensitivity is detailed in the Supplementary Materials.) As shown in Figure 3b, the amplitude of the simulated curve is about 1.28 times that of the measured curve at the feature peak. This difference is attributed to the neglected imaginary part of permittivity in the simulation. The frequency offset between the two characteristic peaks can be ignored. The comparison results mean that the measured curve agrees well with the simulated curve overall. Notably, all samples were tested under the same conditions, and sensing readouts are based on resonant frequency rather than absolute transmittance. This uniform amplitude offset thus introduces no systematic bias in relative comparisons between sample groups.

### 4.2. Discrimination of Suspensions with Different Concentrations

GSCs are a special subset of GBM cells. These cells possess the capabilities of self-renewal, multidirectional differentiation, high tumorigenicity, drug resistance and immune escape. GSCs are recognized as the core seed cells responsible for the initiation, invasion, treatment resistance and recurrence of GBM. Detection of these cells helps clinical diagnosis and tumor classification. Here, a small step was made to discriminate different concentrations of GSC suspension.

Equation (1) indicates that for suspensions with the same type of content, the *ε_eff_* decreases as the concentration *N* rises. In other words, the frequency of the feature peak undergoes a blue shift as concentration *N* increases. GSC suspensions of different concentrations were prepared, and the details are listed in Table 2. 

**Figure 4:**
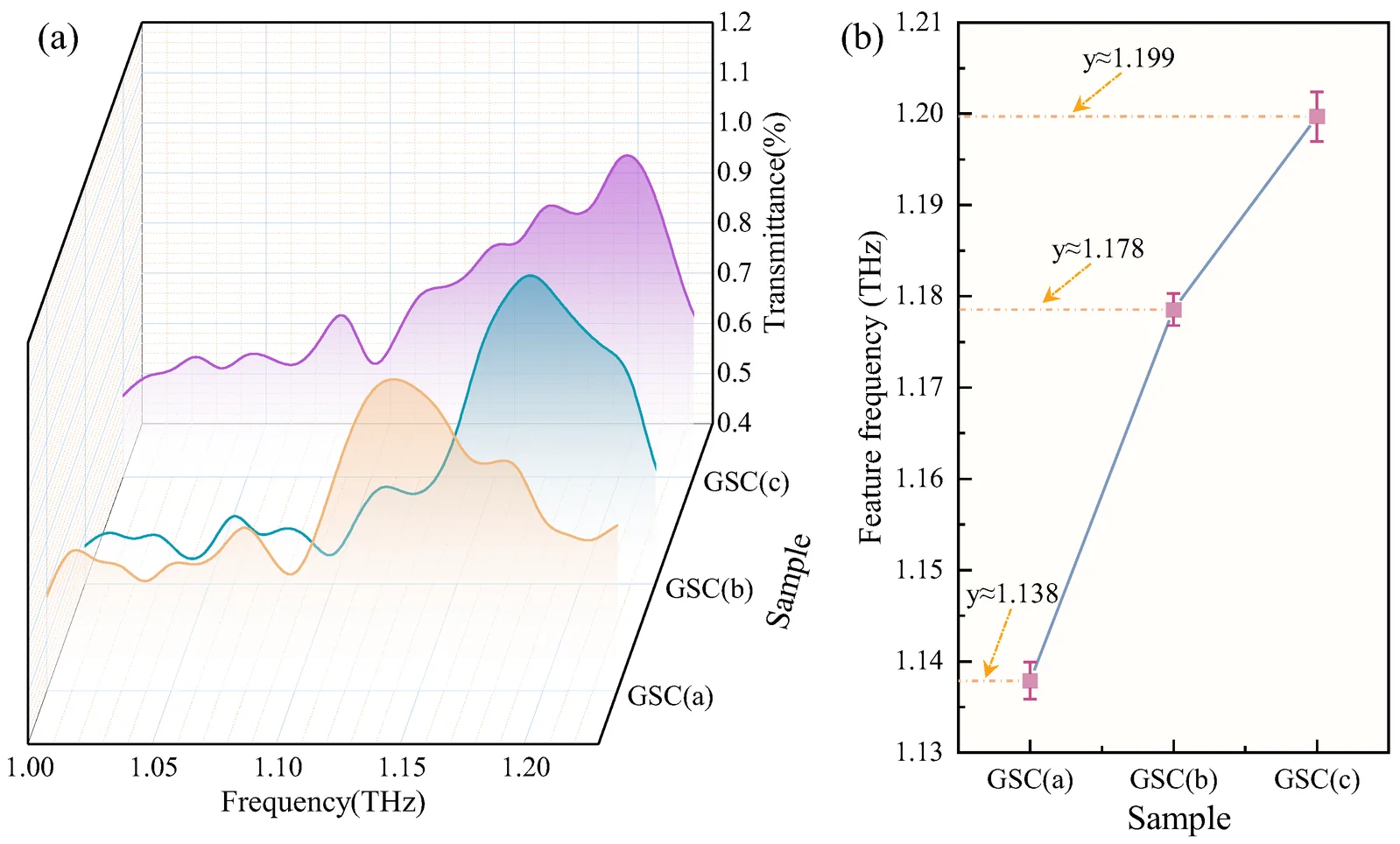
The measurement results for GSC suspension. (**a**) The transmission curves of different cell concentrations. (**b**) The average feature peak resonance frequencies, with error bars extracted from (**a**).

Figure 4a presents transmittance curves of GSC suspension under different concentrations *N*. With the *N* increase from 4 × 10^5^ (S1(a)), 6 × 10^5^ (S1(b)), to 8 × 10^5^ (S1(c)) cells/mL, the feature frequencies shift from ~1.14, ~1.18, and ~1.20 THz, respectively. Furthermore, the imaginary part of *ε_eff_* cannot be neglected in the measurement, which causes the amplitude of the feature peak to increase as *N* increases. The average feature peak frequencies with error bars are shown in Figure 4b. Compared with the feature peak resonance frequency of bare MMs at 1.54 THz, the corresponding frequency shifts Δ*f* are ~380, ~340, and ~320 GHz, respectively. The standard deviation method was employed to quantify measurement errors. The errors for the average feature peak frequencies of S1(a), S1(b) and S1(c) are ~±0.204%, ~±0.176%, and ~±0.271%, respectively.

In the series of suspension S1, the content is GSC cells. The cell type is the same, and the ${\bar{d}}_{\mathrm{GSC}}$ is the same, which indicates that *ε*_S1(a)_ > *ε*_S1(b)_ > *ε*_S1(c)_. In contrast, the feature peak resonance frequency *f*_S1(a)_ < *f*_S1(b)_ < *f*_S1(c)_. Comparing the measurement results with the theoretical inference, both of the feature peak resonance frequencies show as blue shift with the *N* increase. This confirms that the experimental results are consistent with the theoretical inference. Besides, the error bars indicate that the measurement results are reliable; the GSC suspension with a different concentration has been successfully distinguished, which means the liquid biosensor exhibits good performance and can be applied for biological detection.

### 4.3. Distinction of Different Cell Type Suspensions

GSCs are a rare subpopulation of GBM cells, accounting for ~1% of the latter. U87 and U251 are classic differentiated GBM cell lines. Compared with U87 and U251, the GSC possess much stronger stemness, drug resistance and radio resistance. In this study, the GSC samples were isolated from clinical patient specimens, while the U87 and U251 are widely used commercial cell lines. Hence, discrimination of these cell types benefits clinical treatment. Thanks to their inherent biological differences and distinct sources, successful discrimination of these cells benefits optimization of clinical therapeutic strategies against GBM.

**Figure 5:**
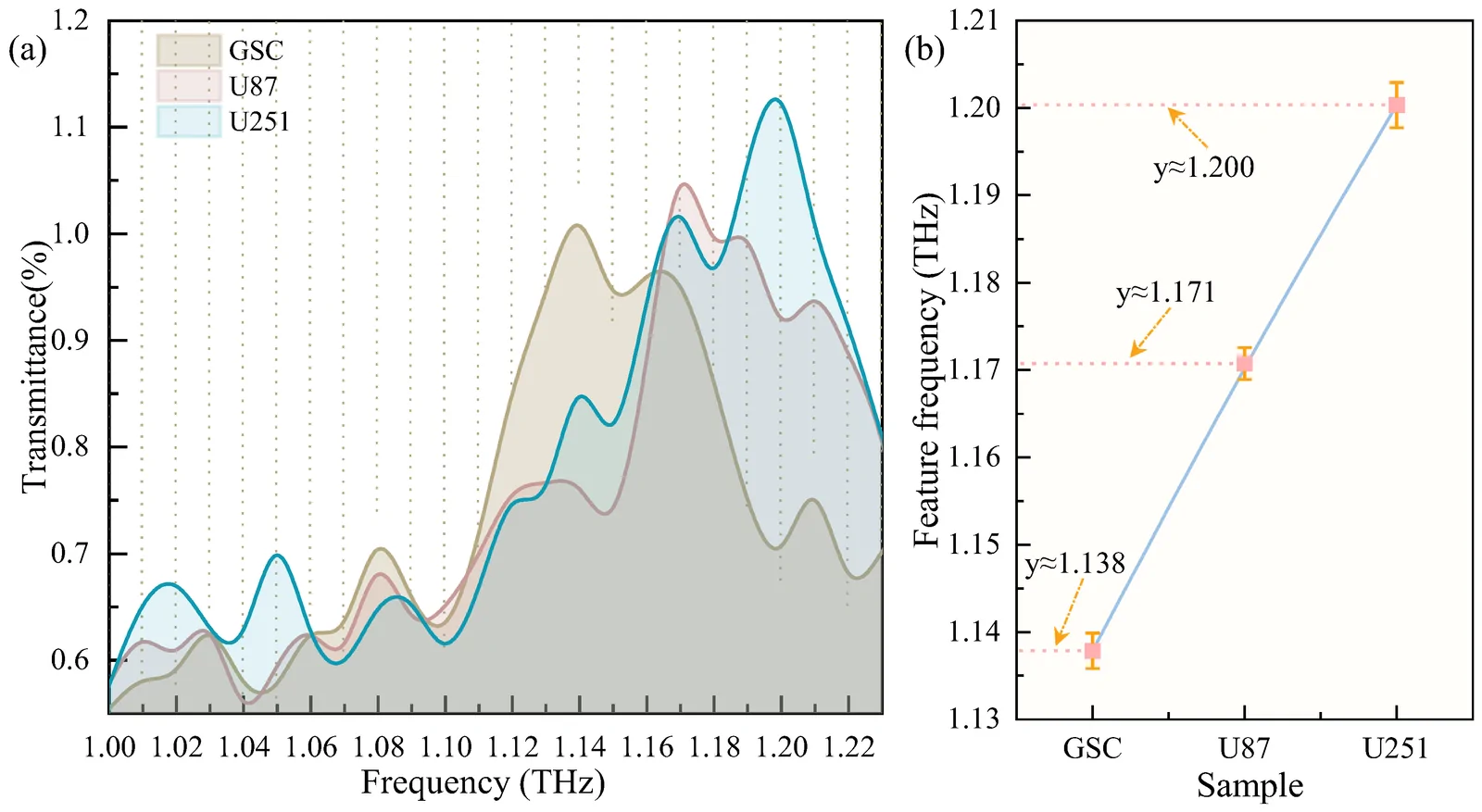
The experimental results for cell discrimination. (**a**) The transmittance curves of GSC, U87, and U251 suspensions under 4 × 10^5^ cells/mL concentration. (**b**) The average feature peak resonance frequencies, with error bars extracted from measured data.

Figure 5 shows the experimental results for cell discrimination. The GSC, U87, and U251 suspensions are under the same concentration *N* = 4 × 10^5^ cells/mL. As shown in Figure 5a, the corresponding feature peak frequencies of the GSC, U87, and U251 suspensions are ~1.14, ~1.17, and ~1.20 THz, respectively. Additionally, Figure 5b demonstrates that the amplitudes of the feature peaks rise with increasing feature peak frequency. The errors of the feature peak frequencies are ~±0.204%, ~±0.183% and ~±0.258%, respectively.

Based on analyses of the trends of feature peak frequency shifts and amplitude rise, and combining Equation (1), the radius relationship among GSC, U87, and U251 should be ${\bar{d}}_{\mathrm{GSC}}$ < ${\bar{d}}_{U87}$ < ${\bar{d}}_{U251}$. Detailed parameters of these suspensions are presented in Table 3.

Details of the cell sizes of GSC, U87 and U251 and the associated measurement methods are provided in Table S1 of the Supplementary Materials [50,51,52,53,54]. The radius relationship of GSC, U87, and U251 agrees with the theoretical inference. Equation (1) reveals that when the concentration N remains unchanged, it should be the case that *ε*_S1_ > *ε*_S2_ > *ε*_S3_. Accordingly, *f*_S1_ < *f*_S2_ < *f*_S3_. As presented in Figure 5b, the experimental results support the theoretical inference. In addition, the error bars indicate that the measurement results are reliable. All results confirm that the liquid biosensor can effectively detect and distinguish the GSC, U87 and U251 suspensions. 

### 4.4. The Serum Suspension Distinction

**Figure 6:**
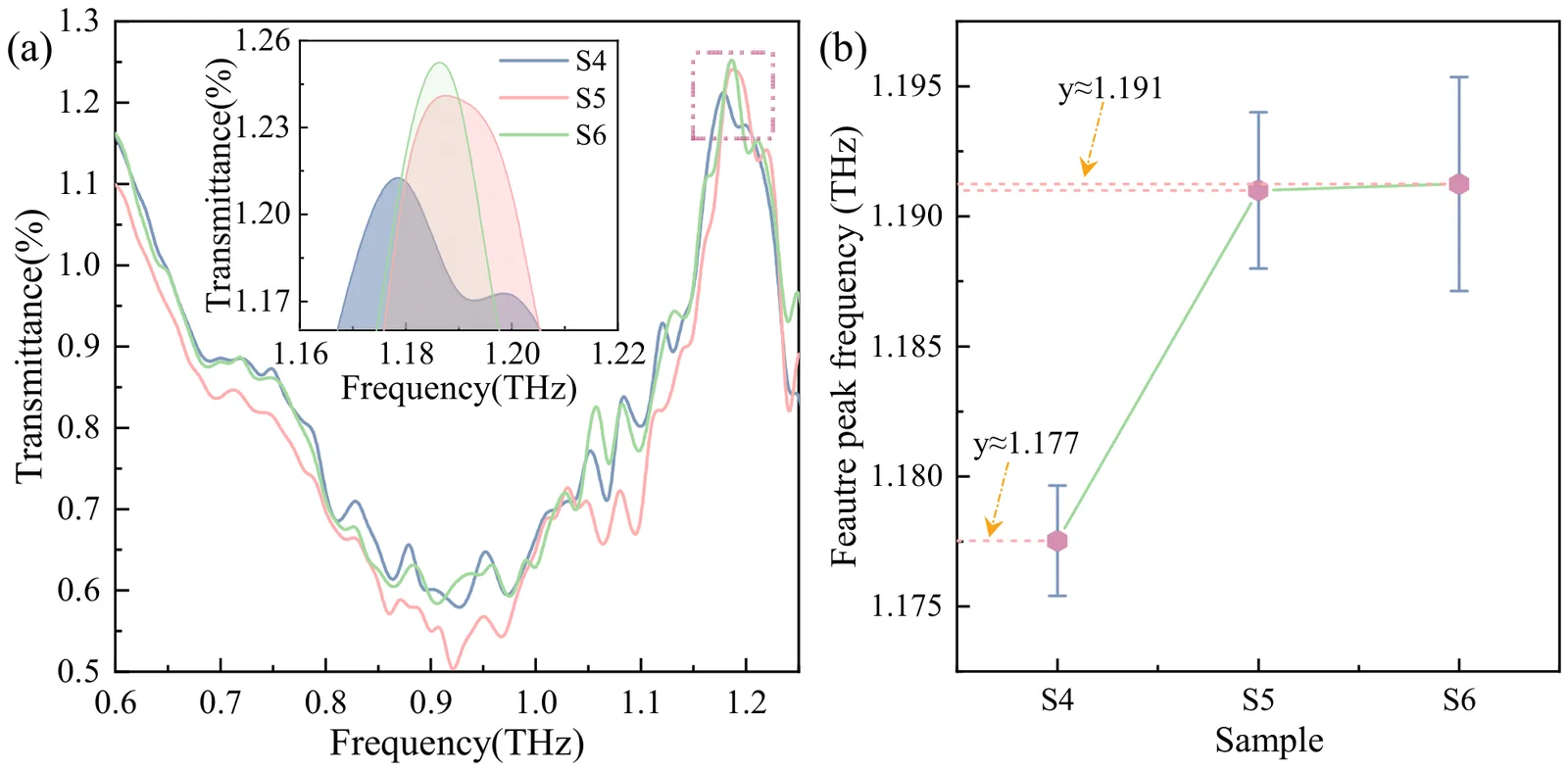
The measurement results for the sera. (**a**) Transmittance curves of samples S4, S5, and S6 (inset: partial enlarged detail of the feature peak region). (**b**) The characteristic peak frequencies with error bars.

Serum from patients with stage IV glioblastoma contains higher levels of tumor-associated proteins, inflammatory cytokines and immune-inflammatory indices, and lower albumin content, compared with serum from healthy individuals. The inflammatory cytokines are kinds of proteins. Immune-inflammatory indices are determined by the counts of neutrophils, lymphocytes, monocytes and platelets in peripheral blood. According to Equation (1), particles within serum suspensions lead to variations in *ε_eff_*. Here, the serum S4 was obtained from a healthy volunteer, while serum samples S5 and S6 were acquired from two glioma patients, respectively. The liquid biosensor is employed to detect serum samples S4, S5, and S6. The measurement results are shown in Figure 6.

Figure 6a presents transmittance curves of serum samples S4, S5, and S6. The jitter on the curves is caused by the optical delay line and the reflection signal, which appear regularly and similarly in the three sample spectra and do not affect the comparative analysis of the characteristic peak. The curves around feature peaks are enlarged to show the details. The curve of S4 can be distinguished from those of S5 and S6. However, the curves of S5 and S6 are difficult to distinguish. The frequencies of feature peaks are shown in Figure 6b. The corresponding feature peak frequencies of S4, S5, and S6 are ~1.18, ~1.19 and ~1.19 THz, respectively. Obviously, serum S4 from the healthy volunteer differs from those of glioma patients. However, S5 and S6, acquired from glioma patients, cannot be discriminated by this liquid biosensor. The relative errors of the feature peak frequencies are ~±0.212%, ~±0.300% and ~±0.411%, respectively. The biosensor has limited capacity for serum discrimination, yet it can still distinguish serum from healthy individuals from that of glioblastoma patients.

## 5. Conclusions

In this study, a low-cost, self-driven THz MMs liquid biosensor is proposed. The liquid biosensor is employed to detect cell suspensions with different concentrations, as well as various types of cell suspensions and serum samples. A microcavity with a thickness of 200 μm and channel-structured MMs were assembled to form the liquid biosensor. Capillary force enables the self-driven infiltration of liquid samples in the biosensor. The resonance peak at ~1.54 THz is selected as the feature peak. The numerically calculated electric field and surface current distributions at ~1.54 THz confirm an electric dipole bright mode. This mode enhances the local electric field and improves sensing performance. The relationships among suspension concentration, particle size, and suspension permittivity have been comprehensively analyzed. The theoretical refractive index sensitivity of the sensor is calculated as 242 GHz/RIU. Different concentrations of GSC suspensions are successfully distinguished by the sensor. Resonance peaks shift from ~1.14, ~1.18, and ~1.20 THz with the rise of cell concentration from 4 × 10^5^, 6 × 10^5^, to 8 × 10^5^ cells/mL, respectively. The experimental results are consistent with the theoretical deduction. GSC, U87 and U251 cell suspensions at the same concentration are also differentiated effectively. The differences in resonance frequencies are matched with the actual cell sizes of three cell types. The corresponding frequencies of feature peaks shift from ~1.14, ~1.17, and ~1.20 THz with increasing cell size, from GSC to U87 and U251. Reliable measurement results are confirmed by low relative errors. Serum samples from healthy volunteers and stage IV glioblastoma patients were detected. Obvious differences are observed between the transmittance curves of healthy and patient serum, with corresponding feature peaks at ~1.18 and ~1.19 THz. However, no effective discrimination is achieved between serum samples from the two glioblastoma patients. The liquid biosensor was proved to have good application potential for the detection of glioma-related cells and serum samples. Overall, the proposed liquid biosensor provides a novel approach for rapid, low-cost cell identification for clinical applications. It also enables effective discrimination of different serum samples. A prominent advantage of this detection platform is that the cellular morphology can be fully maintained during measurement. Furthermore, serum samples only require simple pretreatment before testing.

This work constitutes a methodological device validation study with a limited glioblastoma-related sample cohort, and direct translation to clinical diagnostic practice is not feasible at the present stage. Further study will be advanced along four complementary directions. (a) Machine learning approaches will be integrated to extract multidimensional spectral features for enhanced detection sensitivity. (b) High-sensitivity metamaterial configurations such as bound states in the continuum (BIC) and electromagnetically induced transparency (EIT) structures will be designed to resolve minute permittivity changes in compositionally similar samples. (c) The metamaterial sensing surface will be functionalized with target-specific antibodies to enable specific molecular recognition and detection. (d) The clinical sample cohort will be substantially expanded to encompass healthy controls as well as patients with diverse glioma pathological grades and molecular subtypes, and a corresponding glioblastoma-related sample terahertz spectral database will be established to support calibration and validation of diagnostic thresholds. Through comprehensive enhancements in classification accuracy, sensing sensitivity, detection specificity, and database support, this technology is targeted to be progressively developed as a novel approach for non-invasive sample screening and histopathological grading of glioma.

## Figures and Tables

**Table 1:** The six kinds of suspensions and their content.

Suspension (S)	S1	S2	S3	S4	S5	S6
**Contents**	GSC	U87	U251	Serum1	Serum2	Serum3

**Table 2:** GSC suspensions with different concentrations *N*.

Sample (S1)	S1(a)	S1(b)	S1(c)
Content cells	GSC		
N (cells/mL)	4 × 10^5^	6 × 10^5^	8 × 10^5^
Average radius (μm)	${\bar{d}}_{\mathrm{GSC}}$		

**Table 3:** The detailed information for the GSC, U87, and U251 suspensions.

Sample (S1)	S1	S2	S3
**Content cells**	GSC	U87	U251
***N* (cells/mL)**	4 × 10^5^		
**Average radius (μm)**	${\bar{d}}_{\mathrm{GSC}}$	${\bar{d}}_{U87}$	${\bar{d}}_{U251}$

## Data Availability

Data can be accessed upon reasonable request from the first author, at klchen@buaa.edu.cn.

## Supplementary Materials

The following supporting information can be downloaded at https://www.mdpi.com/article/10.3390/bios16090476/s1, Figure S1: The schematic diagram of microcavity and unit cell of metamaterials. (a) The microcavity structure with dimension label. (b) The unit cells with dimension label; Figure S2: The schematic of the optical modulation angle-resolved THz-TDS image; Table S1: Data of GSC, U87, and U251 and the method for size determination; Video S1: Video of Metamaterial Biosensor Integration.

## Abbreviations

The following abbreviations are used in this manuscript:

MMs	Metamaterials
GSC	Glioblastoma stem cell
THz	Terahertz
PI	Polyimide
Q-BIC	Quasi-bound state in the continuum
RIU	Refractive index unit
BJTTH	Beijing Tiantan Hospital, Capital Medical University
TDS	Time-domain spectroscopy
