# Supplementary material for: Low-Cost Self-Driven Liquid Biosensor Based on Metamaterials for Glioblastoma-Related Sample Detection
Source: Biosensors (Basel). 2026 Aug 30;16(9):476. doi: 10.3390/bios16090476 (PMC13604695; doi:10.3390/bios16090476)
Supplement: Supplementary file 1 [file biosensors-16-00476-s001.zip › biosensors-4427694-supplementary.pdf]

S1. Glioblastoma-related sample approval declaration

Ethical approval for this research protocol was granted by the Ethics Committee of Beijing Tiantan Hospital, Capital Medical University. All serum specimens were collected from patients attending the hospital, who each provided written informed consent prior to sample collection.

S2. Detailed geometric parameters of the unit cell and the microcavity

The parameters of the microcavity are *CL1*=20.00 mm, *CL2*=25 mm, *CG1*=*CL3*= 2 mm, *CL4*=7 mm, *CL5*=13 mm, *CL6*=16 mm, *CL7*=15 mm, *CG2*=1 mm, and *CW*=1 mm, and the thickness of the blue substrate and magenta structural layer is 500 μm and 200 μm， respectively. The unit cell of MMs is shown in Figure 1(c); the blue substrate is quartz (JGS2, China) with a thickness of 500 μm, and the yellow metallic shape is gold with a thickness of 200 nm. The parameters are as follows: *P*=85 μm, *W*=6 μm, *D*= 4 μm, *LR1*=LR4=23.39 μm, *LR2*=*LR3*=7.61 μm, *LR5*=22 μm, *LR6*=41.00 μm, *DR1*=*DL1*=3 μm, *DR2*=*DL2*=2.5 μm, *LL1*=*LL4*=27.19 μm, *LL2*=*LL3*=3.81 μm, *LL5*=42 μm, and *LL6*=41 μm.

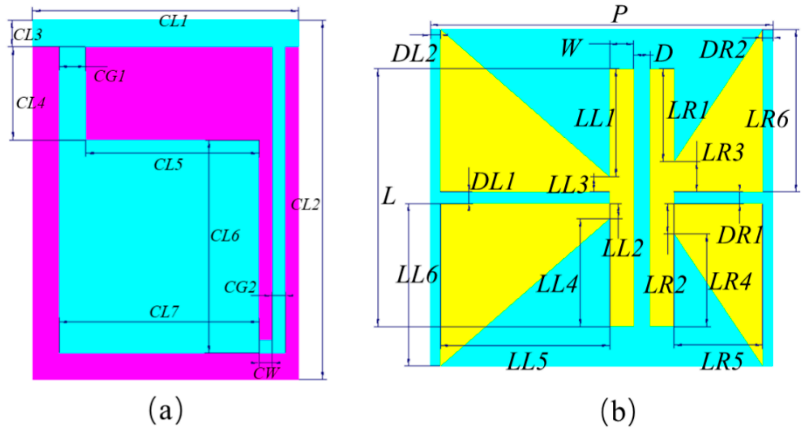

Figure S1. The schematic diagram of microcavity and unit cell of metamaterials. (a) The microcavity structure with dimension label. (b) The unit cells with dimension label.

S3. The cell counting method (NucleoCounter NC-200TM)

The NucleoCounter® NC-200™ is an automated fluorescence-based cell counter. For each sample, the cell pellet was fully resuspended in PBS, and a small aliquot of the homogeneous suspension was withdrawn and loaded into the instrument's proprietary disposable counting cassette. The cassette is preloaded with a nucleic acid fluorescent dye that specifically stains cell nuclei. Fluorescence images are then automatically acquired by the instrument, individual nuclei are identified via its built-in image recognition algorithm, and cell concentration is derived directly. All samples were counted in triplicate, and the mean value was adopted as the final concentration to ensure measurement consistency.

S4. Measurement Setup and Experimental Conditions

The transmission spectrums were measured in self-built THz time-domain spectroscopy (THz-TDS), as shown in Figure 1. The spectroscopy has been mentioned in published papers [1–3].

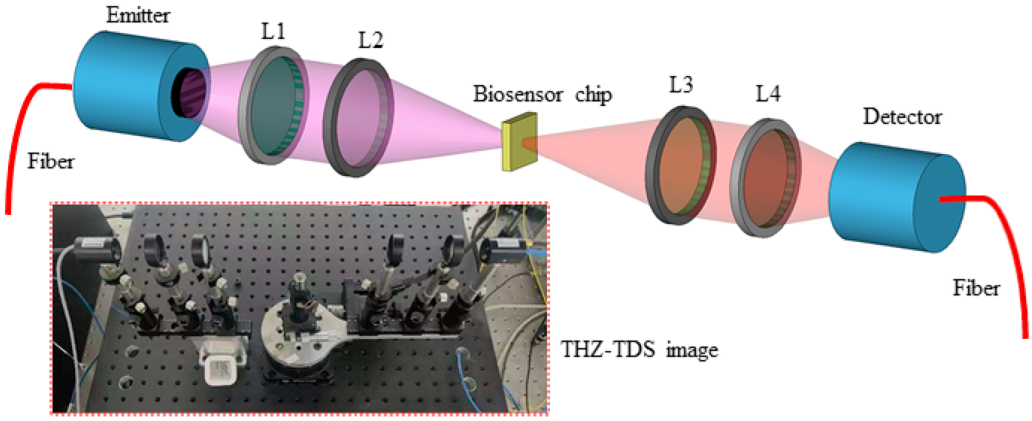

Figure S2. The schematic of the optical modulation angle-resolved THz-TDS image.

The TDS system can generate a horizontal polarization THz wave when the laser drives the InGaAs photoconductive antenna. The laser is generated by a commercial fiber (FemtoFErb FD 6.5, Toptica Company, Germany), whose pulses are 60 fs, spectral width is 1550 nm, and the power is 80 mW. There are two types of lenses shown in Fig. 4, collimating lenses (L1 and L3) with focal length f=50 mm and condenser lenses (L2 and L4) with focal length f=100 mm. Once the THz wave is generated, the wave first comes through L1 and turns to parallel light. Subsequently, the parallel light will focus when it passes through L2, and the sample (biosensor chip) is placed where the focus point is. Then the THz wave, which brings detection messages, passes through L3 and the parallel light propagation to L4. Finally, the THz wave passes through L4 and then focuses on the receiver antenna. The receiver antenna transforms THz radiation into a current signal. The THz radiation electric field vector can be detected by scanning an optical delay line based on phase-locked amplifier technology. The length of the optical delay line is 30 mm; that is the frequency resolution of the THz-TDS system is 5 GHz. The diameter of wave spot size is ~5 mm, and the periodicity of the unit cell is 85 μm, which means ~5000 unit cells can be radiated by THz radiation. The experiment setup and conditions ensure that our measurement results are reliable and can be repeated.

This study's experiments were carried out in Beijing, China, during February. The THz-TDS system was placed in a sterile, temperature- and humidity-controlled room, and measurements were thus conducted under conditions of ~25 °C and~ 30% air humidity.

S5. The fast delay line technique and its potential artifacts

The fast delay line is a common configuration in THz time-domain spectroscopy systems [4,5]. By reducing the mechanical scanning travel of the optical delay line, it substantially shortens the acquisition time for a single spectrum. In this work, the delay line travel was fixed at 30 mm, enabling each sample measurement to be completed within about 50 s. This setting was selected primarily to minimize evaporation of liquid samples in the open microcavity and to suppress ambient temperature drift during prolonged scans, which meets the requirements of rapid clinical screening.

This short-travel scan also introduces measurable artifacts. The narrowed time-domain sampling window cannot fully separate the main transmitted pulse from the multiple Fresnel reflection echoes generated at the quartz substrate and the two interfaces of the microcavity. Following Fourier transform, these residual reflection echoes manifest as periodic amplitude ripples on the frequency-domain transmittance curve, corresponding to the reflection interference noted in the manuscript.

It's worth noting that all cell suspension and serum samples were measured under identical system parameters. This interference artifact acts as a uniform, stable background modulation across all measurements. It only modulates local transmittance amplitude and does not shift the central frequency of the resonant feature peak. Since the sensing readout relies entirely on resonant frequency shift rather than absolute amplitude, this artifact introduces no systematic bias between sample groups. Relative errors in peak frequency across all sample groups are below 0.5%, within the acceptable range of system errors, so the reliability of sample discrimination remains uncompromised.

S6. Details of the cell sizes of GSC, U87 and U251 and the associated measurement methods

Table S1. Data of GSC, U87, and U251 and the method for size determination.

Cell Type	Mean Diameter	Standard Deviation	Method for Size Determination
Undifferentiated GSCs [6]	~10.5 μm	± 1.2 μm	Bright-field single-cell imaging combined with microfluidic deformability cytometry; validated by flow cytometry forward scatter signal intensity—which correlates with cell volume.
U87[7]	13.0 μm	± 1.1 μm	Coulter counter (resistive pulse sensing—the gold-standard method for volumetric measurement of suspended cells)
U251[8]	14.5 μm	± 1.5 μm	Multicellular spheroid volume back-calculation (under identical seeding density and culture duration), combined with head-to-head morphological comparison with U87.

S7. The details regarding the impact of ripple waves on sensor sensitivity

The impact of these ripples on sensor performance is limited, and their effect on sensitivity is clarified as below:

(A) The intrinsic sensitivity of the device remains entirely unaffected. The theoretical value of 242 GHz/RIU characterizes the shift in the central frequency of the electric dipole resonance per unit change in environmental refractive index, which is an intrinsic property determined solely by the metamaterial and the nature of the resonant mode. The FP ripples act purely as a periodic amplitude modulation superimposed on the underlying transmission envelope. They neither displace the central frequency of the main resonance nor alter the linear relationship between refractive index variation and resonant frequency shift.

(B) These ripples cause only a minor reduction in peak extraction precision and do not limit practical detection performance. The FP oscillation period is far narrower than the overall linewidth of the resonant envelope, and our 5 GHz

frequency sampling resolution captures the ripple pattern with high fidelity. By fitting the resonant region to a Lorentzian or Gaussian envelope, the periodic ripple structure can be effectively smoothed out. As quantified by our experimental results, the relative standard deviation of the measured peak frequencies across repeated measurements is only ±0.176% to ±0.411%, corresponding to an absolute frequency uncertainty of approximately 2–5 GHz. This is substantially smaller than the frequency separations between biologically distinct sample groups: 20–60 GHz between GSC suspensions of different concentrations, ~30 GHz among the GSC, U87 and U251 cell lines at matched cell density, and a clear frequency gap between serum from healthy donors and patient samples. Therefore, the FP ripples do not impede reliable sample discrimination.

(C) All samples are measured sequentially on the same assembled sensor under identical experimental conditions. As a common-mode artifact, the FP interference pattern remains perfectly stable across all measurements. When comparing relative frequency shifts between samples, this shared modulation cancels out almost completely, rendering relative shift measurements highly robust.

S8. The effect of the imaginary part of ε_w on sensitivity

The impact of ripple waves on sensor sensitivity is as follows:

(A) The core metric adopted in our simulation is the resonant frequency shift in the metamaterial, rather than the absolute transmission amplitude. In accordance with fundamental electromagnetic resonance theory, the central frequency of the electric dipole resonance is predominantly determined by the structural geometry and the real part of the surrounding permittivity, which defines the phase-matching condition of the resonant mode. The imaginary part of permittivity, which represents dielectric loss, primarily attenuates field intensity, reduces the resonant quality factor, and lowers the transmission amplitude. Its perturbation to the central resonant frequency is a high-order effect that is negligible for simulation-level design and trend analysis. This simplification therefore introduces only a systematic amplitude offset, and does not distort the frequency shift trend or the physical law of dielectric loading.

(B) This simplification does not compromise the validity of the theoretical sensitivity calculation. The theoretical sensitivity is defined as the resonance frequency shift per unit change in the real part of the refractive index ($S=\Delta f/\Delta n$), a metric that characterizes the linear response of resonant frequency to real-index loading. Incorporating dielectric loss reduces the simulated peak amplitude and Q-factor, yet does not materially alter the linear scaling relationship between Δn and Δf. As an intrinsic dispersive property of the metamaterial structure, frequency-shift sensitivity is overwhelmingly dominated by the real part of environmental permittivity.

(C) Full implementation of water's frequency-dependent complex permittivity would require full-spectrum dielectric dispersion modeling and substantially increase simulation complexity, yet would add no meaningful insight to the core contributions of this work. The innovations of this study lie in the channel-structured metamaterial design and the theoretical analysis of suspension effective permittivity, neither of which relies on precise amplitude matching in simulations. The 1.28 times amplitude discrepancy is an expected consequence of omitted absorption loss; this uniform amplitude scaling effect does not alter relative frequency differences under different dielectric loading conditions.

S9. Detailed information of the PBS solution

All cell washing procedures in this study were performed with standard 1×phosphate-buffered saline (PBS) adjusted to pH 7.2–7.4. This formulation is universally adopted for mammalian cell handling, with osmolarity and pH matched to physiological conditions to preserve cell morphology and viability during rinsing. The standard 1× working solution consists of 137 mM NaCl, 2.7 mM KCl, 10 mM Na_2HPO_4, and 2 mM KH_2PO_4; the same batch of PBS was used throughout all sample preparation steps to ensure consistency.

Reference

1. Han, L.; Wang, Y.; Chen, K.; Gao, H.; Xia, K.; Ge, Q.; Yang, J.; Shi, W.; Ruan, C. Detection of In Vivo-like Cells by a Biosensor Chip Based on Metamaterials in Terahertz Regime. *Biosens.* **2024**, *14*, 230. https://doi.org/10.3390/bios14050230.
2. Yang, P. D.; Ouyang, C.; Hong, T. S.; Zhang, W. H.; Miao, J. G.; Wu, X. J. Study of phase transition of single crystal and polycrystalline vanadium dioxide nanofilms by using continuous laser pump-terahertz probe technique. *Acta Phys. Sin.* **2020**, *69*, 204205. https://doi.org/10.7498/aps.69.20201188.
3. Xiong, H.; Sun, H.; Zhou, J.; Li, H.; Zhang, H.; Liu, S.; Cai, J.; Feng, L.; Miao, J.; Chen, S.; et al. Terahertz Anisotropy in Fascia and Lean Meat Tissues. *Biomed. Opt. Express* **2022**, *13*, 2605–2615. https://doi.org/10.1364/BOE.454338.
4. Hoberg, C.; Balzerowski, P.; Havenith, M. Integration of a Rapid Scanning Technique into THz Time-Domain Spectrometers for Nonlinear THz Spect roscopy Measurements. *AIP Advances*, **2019**, *9*, 035348.
5. Wang, Q.; Xie, L.; Ying, Y. Overview of Imaging Methods Based on Terahertz Time-Domain Spectroscopy. *Applied Spectroscopy Reviews* **2022**, *57*, 249–264. https://doi.org/10.1080/05704928.2021.1875480.
6. Asghari, M.; Ivetich, S.D.; Aslan, M.K.; Aramesh, M.; Melkonyan, O.; Meng, Y.; Xu, R.; Colombo, M.; Weiss, T.; Balabanov, S.; et al. Real-Time Viscoelastic Deformability Cytometry: High-Throughput Mechanical Phenotyping of Liquid and Solid Biopsies. *Sci. Adv.* **2024**, *10*, eabj1133. https://doi.org/10.1126/sciadv.abj1133.
7. Oraiopoulou, M.-E.; Tzamali, E.; Tzedakis, G.; Vakis, A.; Papamatheakis, J.; Sakkalis, V. *In Vitro/In Silico* Study on the Role of Doubling Time Heterogeneity among Primary Glioblastoma Cell Lines. *BioMed Research International* **2017**, *2017*, 1–12. https://doi.org/10.1155/2017/8569328.
8. Wanigasekara, J.; Carroll, L.J.; Cullen, P.J.; Tiwari, B.; Curtin, J.F. Three-Dimensional (3D) in Vitro Cell Culture Protocols to Enhance Glioblastoma Research. *PLoS ONE* **2023**, *18*, e0276248. https://doi.org/10.1371/journal.pone.0276248.
